# Design and Assessment of a Polyurethane Carrier Used for the Transmembrane Transfer of Acyclovir

**DOI:** 10.3390/nano11010051

**Published:** 2020-12-28

**Authors:** Florin Borcan, Adél Len, Cristina A. Dehelean, Zoltán Dudás, Roxana Ghiulai, Andrada Iftode, Roxana Racoviceanu, Codruta M. Soica

**Affiliations:** 1 Department I, Faculty of Pharmacy, “Victor Babes” University of Medicine and Pharmacy, 300041 Timisoara, Romania; fborcan@umft.ro; 2 Neutron Spectroscopy Department, Centre for Energy Research, H-1121 Budapest, Hungary; len.adel@ek-cer.hu; 3 Faculty of Engineering and Information Technology, University of Pécs, H-7624 Pécs, Hungary; 4 Department II, Faculty of Pharmacy, “Victor Babes” University of Medicine and Pharmacy, 300041 Timisoara, Romania; cadehelean@umft.ro (C.A.D.); andradaiftode@umft.ro (A.I.); babuta.roxana@umft.ro (R.R.); codrutasoica@umft.ro (C.M.S.); 5 “Coriolan Drăgulescu” Institute of Chemistry, 300223 Timisoara, Romania

**Keywords:** drug delivery, DSC, FT-IR, SANS, TEWA, Zeta potential

## Abstract

The Herpes simplex viruses (HSV-1, HSV-2) are responsible for a wide variety of conditions, from cutaneous-mucosal to central nervous system (CNS) infections and occasional infections of the visceral organs, some of them with a lethal end. Acyclovir is often used intravenously, orally, or locally to treat herpetic infections but it must be administered with caution to patients with kidney disease and to children of early age. The main objectives of this study were to synthesize and evaluate new polyurethane nanoparticles that might be used as proper transmembrane carriers for acyclovir. Polyurethane particles were obtained by a polyaddition process: a mixture of two aliphatic diisocyanates used as organic phase was added to a mixture of butanediol and polyethylene glycol used as aqueous phase. Two different samples (with and without acyclovir, respectively) were synthesized and characterized by UV-Vis spectra in order to assess the encapsulation efficacy and the release profile, FT-IR, DSC, SEM, and SANS for structural characterization, as well as skin irritation tests. Nearly homogeneous samples with particle sizes between 78 and 91 nm have been prepared and characterized revealing a medium tendency to form clusters and a high resistance to heat up to 300 °C. The release profile of these nanoparticles is characteristic to a drug delivery system with a late discharge of the loaded active agents. Very slight increases in the level of transepidermal water loss and erythema were found in a 15-day evaluation on human skin. The results suggest the synthesis of a non-irritative carrier with a high encapsulation efficacy that can be successfully used for the transmembrane transfer of acyclovir.

## 1. Introduction

**Figure 1:**
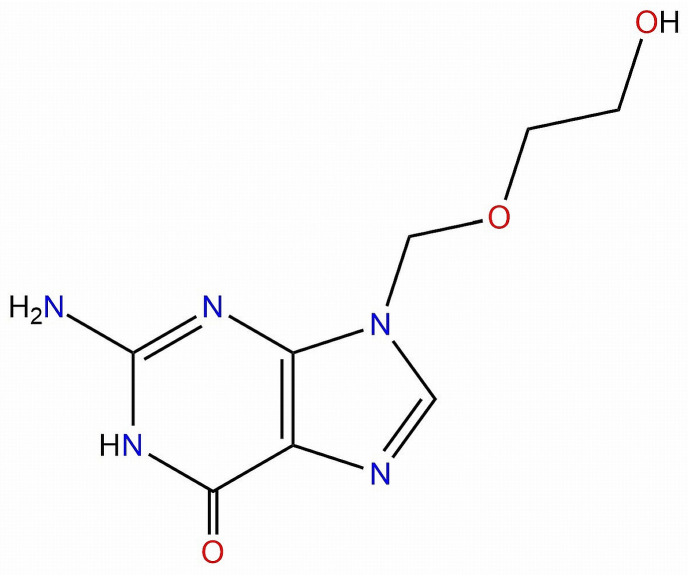
The chemical structure of acyclovir.

Acyclovir (Figure 1) is a guanosine synthetic analogue widely used in the treatment of herpetic infections, particularly Herpes simplex and Varicella-zoster viruses; its action is based on its bioactivation to acyclovir monophosphate and further to acyclovir triphosphate which acts as a competitive inhibitor of DNA polymerase due to its similarity to the structure of deoxyguanosine triphosphate [1].

Acyclovir is mainly excreted unchanged in the urine; therefore, the associated medication eliminated by the same mechanism may delay its elimination and increase its plasmatic concentration [2]. Acyclovir should be administered with caution in patients with renal impairment (creatinine clearance below 10 mL/min) especially in elderly patients to whom an adequate hydration must be ensured. The risk of renal failure is increased by the use of other nephrotoxic drugs [3]. The following side effects were often reported: headache, dizziness, pruritus, transient rashes, nausea, vomiting, diarrhea, and abdominal pain [4,5]. In order to overcome these side effects and to accomplish a targeted drug release acyclovir can be embedded in a solid support such as polyurethane nanoparticles.

The synthesis of biocompatible, pH-responsive and biodegradable polyurethanes as smart delivery systems, using safe, cheap and easily available amino acids as chain extenders, was described by M. Shoaib et al. [6]. They found an enhanced release rate of imatinib due to the fast degradation of polycaprolactone used as the main component of the hydroxylic phase followed by strong in vitro and in vivo antitumor effects as suggested by MTT assay data and histopathological evaluations on BALB/c mice, respectively. The same research team have reported another study which combined mesoporous bioactive glass with polyurethanes thus creating a nanocomposite capable to provide a sustained imatinib release [7]. The mesoporous bioactive glass was used as filler, while the biocompatible polyurethane provided the matrix material for the development of bioactive imatinib-loaded nanocomposites with non-cytotoxic profile as indicated by a high cell viability (96–100%) as well as high cumulative drug release (52–84%) over a period of three weeks.

Solid nanoparticles based on carboxylated cyclodextrin sponges have been developed by D. Lembo et al. as an acyclovir delivery system [8]. They found that the carboxylated sample has encapsulated a higher amount of the active agent than the un-carboxylated analogue and exhibited an increased antiviral activity against the clinically isolated Herpes simplex virus HSV-1. An alternative enhancement of its antiviral activity was found after the co-encapsulation of acyclovir and curcumin inside polymeric microparticles [9].

The relevance of acyclovir carriers’ development has been emphasized by Hassan et al. [10] who described various acyclovir administration routes and their respective disadvantages as well as novel drug delivery systems such as liposomes, poly(ethylene glycol)-co-cyclic acetal and polylactic acid nanospheres coated with polyethylene glycol.

The choice of a proper strategy for drug administration plays a crucial role in the development of new therapeutic systems. Polyurethanes (PU) versatility is provided by the fact that they can be used in almost all types of delivery systems (transdermal, oral, etc.); as an example, PU dendrimers have been developed as drug carriers for various pharmaceutical agents such as the stable nitroxide free radical 2,2,6,6-tetramethylpiperidinyl-1-oxy (TEMPO) [11,12]. PU are described as safe, biodegradable and haemocompatible blood-contacting materials. They are already used as prostheses for substitution of the cardiac valve [13], in the hybrid artificial pancreas with a dense polyurethane membrane [14], etc. Knowing that the urethane bond is the most unstable in these products in any degradation process, polyols and isocyanates are obtained in PU degradation [15], but it is necessary to mention that isocyanate groups are rapidly converted in amine inside any aqueous medium. K.S. Brockman et al. [16] have found that PU present a minimal haemocompatibility changes over their degradation, while B. van Minnen et al. [17] have found that a PU foam can be safely used as a biodegradable implant in a three-year subcutaneous implantation study

In our previous studies, polyurethane nano- and micro-particles were synthesized and characterized as transmembrane delivery systems for different active agents [18,19,20,21]. Until recently no studies were conducted regarding the use of polyurethane-based materials for the controlled delivery of acyclovir, probably because the best research and development teams and the majority of patents in polyurethanes are in different industrial sectors (automotive industry, the insulation applications, furniture, camping products etc.) due to the global market growth in these fields (USD 41.83 Billion in 2015 and an expected USD 56.76 Billion next year according to an official report [22]). Therefore, the main aim of this research was the design and assessment of PU carriers that can ensure the prolonged and sustained release of acyclovir; the novelty and originality of the current study consists in the synthesis of PU nanoparticles that was conducted in the absence of any catalyst or polymerization initiator, with a decreased amount of surfactant.

## 2. Materials and Methods

### 2.1. Reagents

Hexamethylene diisocyanate (HDI), isophorone diisocyanate (IPDI) and polyethylene glycol (PEG 200) were purchased from Merck (Darmstadt, Germany), acetone from Chimopar SA (Bucharest, Romania) and 1,4-butanediol (BD) from Carl Roth GmbH (Karlsruhe, Germany). Different chlorides, phosphates and carbonates such as NaCl, KCl and MgCl_2_, Na_2_HPO_4_, K_2_HPO_4_, KH_2_PO_4_ and NaHCO_3_ were acquired from Chimopar SA (Bucharest, Romania) and used to prepare the simulated body fluid (SBF).

### 2.2. Synthesis of the PU Particles

In the first step, an aqueous phase (a mixture of 1.6 mL BD, 3.4 mL PEG 200, and 65.0 mL deionized water) was sonicated with a lab homogenizer Hielscher UP200S (Teltow, Germany) for 20 min and then split in two separate 100 mL flasks in equal volumes; 3.0 mL acyclovir aqueous solution (1.0 mg/mL) was added in the first flask (the active sample, PU_NP_acycl), while 3.0 mL deionized water was poured in the second flask (the reference sample, PU_NP_control). In parallel, an organic phase (a mixture of 1.8 mL HDI, 1.2 mL IPDI and 27 mL acetone) was mixed on a hot plate stirrer Velp (Usmate, Italy) at 350 rpm and 45 °C for 20 min.

In the second step, half of the organic mixture was rapidly injected in the PU_NP_acycl flask, while the other half in the PU_NP_control flask. The contents of the flasks were then stirred at 350 rpm and room temperature for 3 h to complete the synthesis of the macromolecular chains.

Repetitive cycles of washing-centrifugation of the white suspensions have been performed three times using a mixture of acetone and water (1:1, v/v). Finally, the two samples were slowly dried in thin layers at room temperature and normal pressure until constant mass.

### 2.3. Drug Loading

The percentage of acyclovir successfully entrapped inside the PU particles was calculated by reporting the free amount of drug to the total added amount. The free acyclovir amount was calculated using the Beer–Lambert law at 470 nm in accordance with S.A. Reddy et al. [23] and a UVi Line 9400 Spectrophotometer SI Analytics (Mainz, Germany). The amount of free acyclovir collected in the washing mixture resulted from the last step of the synthesis, was calculated based on a method previously reported in the literature which allowed the construction of a calibration curve by plotting the absorption values as a function of drug concentration, described by Equation (1) [23]:

y = 0.1136x, R^2^ = 0.9987 (1)

where: 

y = absorption value

x = drug concentration (µg/mL)

R^2^ = the coefficient of determination

### 2.4. Drug Release Profile

The cumulative release percentage has been assessed by maintaining the PU particles with acyclovir (sample PU_NP_acycl) in a simulated body fluid (SBF), prepared according to T. Kokubo recipe [24] for 15 days; the procedure was described in one of our previous papers [19]. Briefly, 2.0 mL aqueous solution of PU_NP_acycl sample (1.0 mg/mL) were placed in 30 mL SBF and 3 aliquots of each 0.5 mL were replaced every third day with fresh medium; their maximum absorption was measured at 470 nm and mean concentrations ± SD were used to explain the release profile.

### 2.5. Dissolution Profile

An in vitro dissolution investigation was run using a modified Erweka DT instrument (Erweka, Langen, Germany), at 37 ± 2 °C, in 100 mL simulated gastric/intestinal medium, at 100 rpm. 5 mL samples were collected in each case at ¼, ½, 1, 2, 4, 6, 8, 10, 18 and 24 h to evaluate the release profile of acyclovir. Briefly, the concentration of acyclovir was assessed spectrophotometrically by means of an UVi Line 9400 Spectrophotometer SI Analytics (Mainz, Germany) using calibration curves for acyclovir for each dissolution medium; the compositions of simulated gastric/intestinal medium were already described in one of our previous papers [25]. All the measurements were conducted in triplicate in order to gain statistical value. The dependence between the amount of solubilized acyclovir and the UV absorption in the simulated media is described by Equations (2) and (3):

y(g) = 0.0917x (R^2^ = 0.9914) (2)

y(i) = 0.0738x (R^2^ = 0.9971) (3)

### 2.6. The Penetrability Rate

The capacity to penetrate through different membranes was tested using a PVDF artificial membrane Spectra/Por^®^ and the osmosis process, a procedure described in the literature [18]. 2.0 mL tested sample was loaded inside the dialysis tube and 100 mL saline buffer were used in the external chamber. The experiment was done at 25 ± 1 °C, and at every 10 h, 1.0 mL liquid from the external chamber was replaced with fresh buffer and it was analyzed spectrophotometrically at 290 nm.

### 2.7. Thermal Analysis

The two samples and pure acyclovir, respectively, have been thermally degraded using a DSC1 Mettler-Toledo (Greifensee, Switzerland); small amounts (4 ± 0.4 mg) were placed in aluminum crucibles with pierced lids and then heated under inert atmosphere between 30 and 300 °C with 5 °C/min.

### 2.8. Zetasizer Tests

The size and the surface charge of the particles from the two samples have been evaluated using a Zetasizer module Cordouan Technol. (Pessac, France). The following parameters have been set for the determination of particle size: temperature (22 ± 1 °C); time intervals (10 ± 3 μs); number of channels (250 ± 10); laser power (75 ± 5%); acquisition mode (statistical mode with noise limit; minimum 3 acquisition/sample) and analysis mode (Cumulants). The parameters for the Zeta potentials were: quartz cuvette; temperature (22 ± 1 °C); laser power (75 ± 5%); applied field (automatic); medium resolution; 3 measures/sequence and Henry function (Smoluchowski).

### 2.9. Scanning Electron Microscopy (SEM)

SEM images of the samples were recorded by a TESCAN 3 VEGA scanning electron microscope secondary electron detector (Brno, Czech Republic). The accelerating voltages value was set at 20 kV and the magnifications to 5000× and 1000×.

### 2.10. FT-IR Spectroscopy

Infrared spectra were recorded in the range of 400–4000 cm^−1^ using a Cary 630 FTIR instrument, using KBr pellets. The resolution was 4 cm^−1^ and 64 scans per measurement were performed.

### 2.11. Small-Angle Neutron Scattering (SANS)

Dry powder samples were introduced in 2 mm thick quartz cuvettes, then placed in the neutron beam of the SANS instrument, Yellow Submarine at Budapest Neutron Centre and measured at room temperature. Yellow Submarine is a pin-hole type instrument, with a two-dimension neutron detector. Two wavelengths (4.15 Å and 9.7 Å) were used at a sample-to-detector distance of 5.2 m. This set-up covers a Q (scattering vector) range of 0.008 Å^−1^ to 0.1 Å^−1^, the scattering vector being defined as Equation (4):

(4) $$Q=\frac{4\pi }{\lambda }sin\frac{\theta }{2}$$

where *λ* is the wavelength, and *θ* is the scattering angle.

The scattered neutron intensity was calibrated by instrumental noise, scattering from the empty cell, detector pixel sensitivity, and sample volume. The transmission of the samples has been considered. For samples where the scattering objects (pores, voids, inhomogeneities, nano sized particles) orientation is random, the SANS intensity is isotropic, therefore a radial averaging over the same scattering angles can be done, resulting in a curve which shows the modulation of the scattered intensity versus the Q scattering vector. The SANS curves are then evaluated with the aid of mathematical models, describing the structural characteristics of the samples. In the present case, a simple approximation has been used and the least squared algorithm fits were done with a power law model, per Equation (5).

(5) $$I\left(Q\right)=A\cdot Q^{-p}$$

where *I(Q)* is the corrected neutron scattering intensity measured on the detector, *A* is an adjustable scaling parameter, and *p* is the power-law exponent [26].

In the measured *Q* region, the value of the power-law exponent characterizes the scattering interfaces, and it can be converted into fractal dimensions.

### 2.12. Skin Irritation Evaluations

Six healthy human volunteers (2 men and 4 women, between 31 and 42 years old) were subjected to the topical application of samples with or without acyclovir, respectively, in order to further evaluate the skin irritation induced by the use of each product. The two synthesized samples (PU_NP_control and PU_NP_acycl) have been applied as aqueous solutions (1:20 w/v) on different areas of the left anterior forearm every third day (0.5 mL/application); the assessment of skin irritation has been conducted 20 min later on each area. The tests were carried out with a MultiProbe Adapter System from Courage & Khazaka Electronics (Koln, Germany), equipped with a Tewameter^®^ TM300 probe and a Mexameter^®^ MX18 probe. Average values calculated as differences between the values recorded before and after application were collected, taking into account the variations in skin type between the investigated volunteers.

### 2.13. Statistics and Ethical Approval

The statistical analyses were performed using the IBM SPSS v.27; one-way ANOVA and Bonferonni–Dunn tests were used to determine the statistical difference between the two experimental groups; ** and *** indicate *p* < 0.01 and *p* < 0.001 values.

The research was conducted according to the principles of the Declaration of Helsinki. Authors declare that all procedures followed the specific regulations and standards; the study was evaluated and approved by the Ethical Committee of University of Medicine and Pharmacy Timisoara, Romania (Approval no. 16/2020). All volunteers have read and signed informed consents regarding the applied experimental procedures.

## 3. Results

### 3.1. Drug Loading Efficacy

The maximum absorption peak of the acyclovir-loaded PU nanoparticles was recorded at 310 nm, while the maximum absorption peak of pure acyclovir was found at 470 nm. The drug loading efficacy expressed as the ratio between the free amount of acyclovir calculated according to Equation (1) and the total amount of the drug that entered the synthesis reached 77.9%.

### 3.2. Drug Release Profile

**Figure 2:**
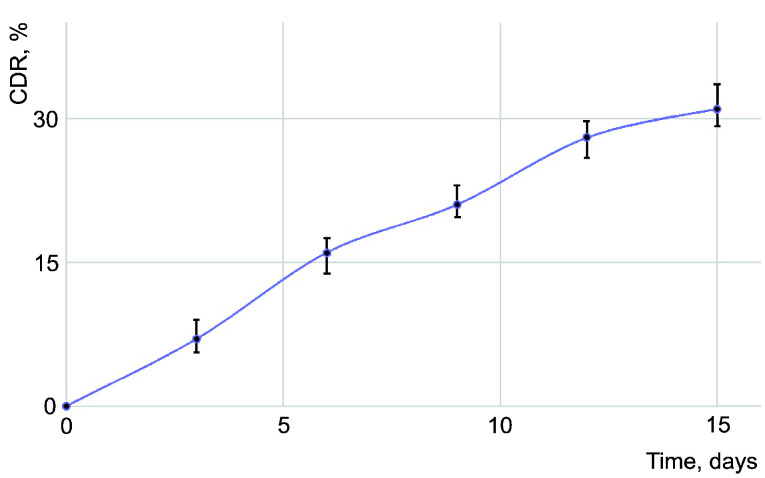
The cumulative drug release (CDR) of acyclovir from PU_NP_acycl sample.

An almost linear increase of the cumulative percentage of the acyclovir released inside the simulated body fluid was obtained by maintaining 2 mL aqueous solution of PU_NP_acycl sample (1.0 mg/mL) in 30 mL SBF (Figure 2). SBF maximum absorption was measured at 470 nm every third day using pure SBF as reference.

### 3.3. Dissolution Tests

**Figure 3:**
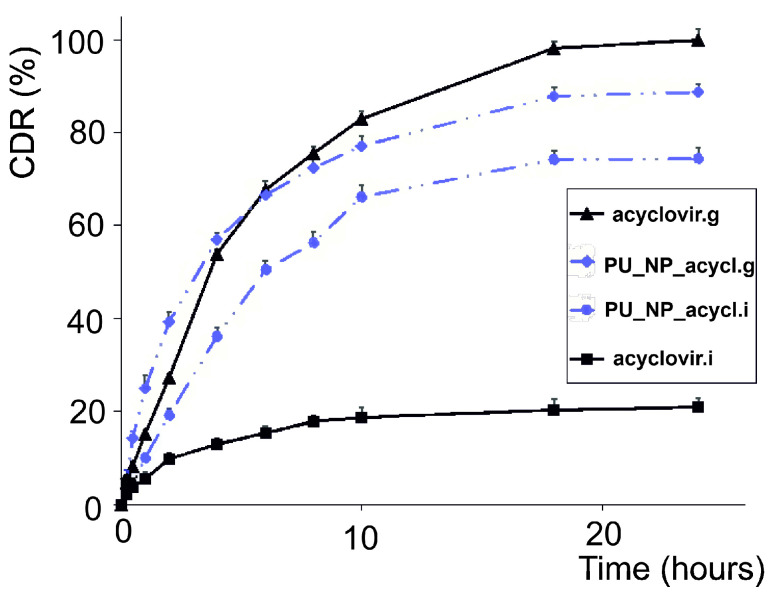
In vitro dissolution profiles in simulated gastric (g)/intestinal (i) media.

The dissolution behavior of pure acyclovir and PU_NP_acycl sample was graphically represented in each medium (Figure 3) based on the Equations (2) and (3) and the Beer–Lambert law.

In vitro dissolution tests showed that acyclovir solubilizes poorly in neutral environment (simulated intestinal medium, pH = 7); a dramatic solubility increase was recorded in acid medium (simulated gastric medium, pH = 1.2), emphasizing the strong pH-dependency of acyclovir solubility. Following PU entrapment, the dissolution profile of acyclovir improves in intestinal neutral medium; in gastric environment, the dissolution profile changes shape and reveals a lower amount of released drug over 24 h, without the initial burst release noticeable for pure acyclovir. The dissolution curves of acyclovir-loaded PU particles in gastric and intestinal media indicate a sustained release of the drug. However, the cumulative drug release in gastric medium is higher compared to the intestinal medium, behavior that may be pharmacologically significant.

### 3.4. The Penetration of Membranes

**Figure 4:**
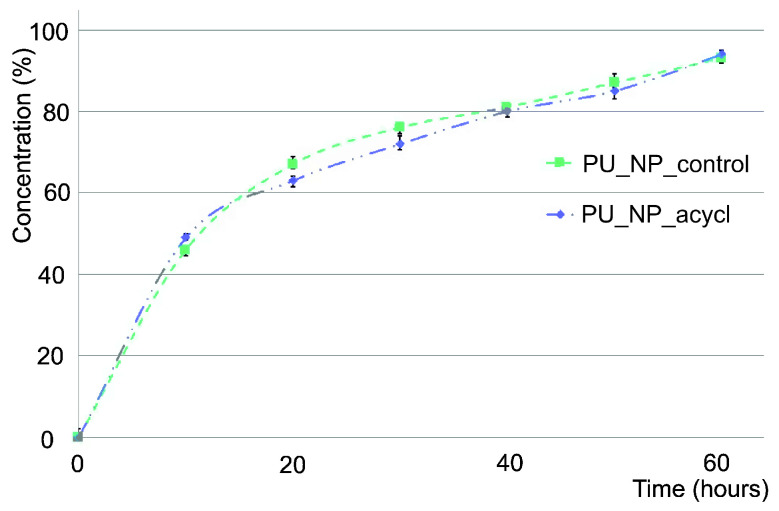
The membranes’ penetration rates.

Fast penetration processes were observed for both tested samples (PU_NP_control and PU_NP_acycl (Figure 4). It can be observed that almost 50% of particles penetrated the artificial membrane in the first ten hours and almost 75% passed through the membrane in the first 24 h.

### 3.5. Thermal Analysis

**Figure 5:**
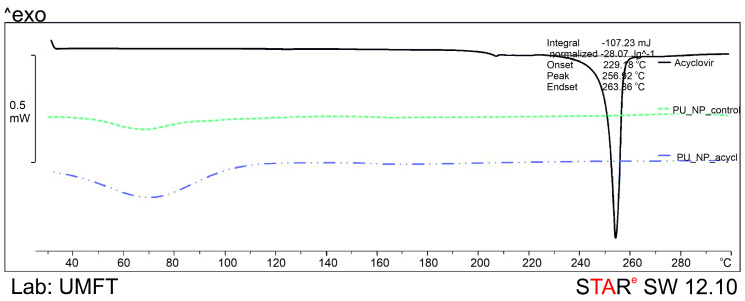
DSC thermograms of tested samples.

The melting point of pure acyclovir (around 256 °C as described in the literature [27]) accompanied by a slight solvent evaporation was reported between 40 and 100 °C on the DSC thermograms (Figure 5).

### 3.6. Zetasizer Tests

The distribution of PU particle size and their surface charge are reported in Table 1. The values are specific to almost homogeneous samples with a medium tendency to agglomerate.

### 3.7. Scanning Electron Microscopy (SEM)

**Figure 6:**
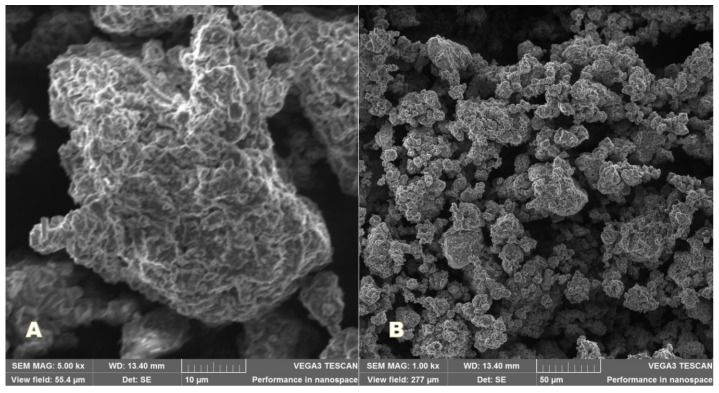
SEM image of PU particles in PU_NP_acycl sample: (**A**) 5000×; (**B**) 1000×.

**Figure 7:**
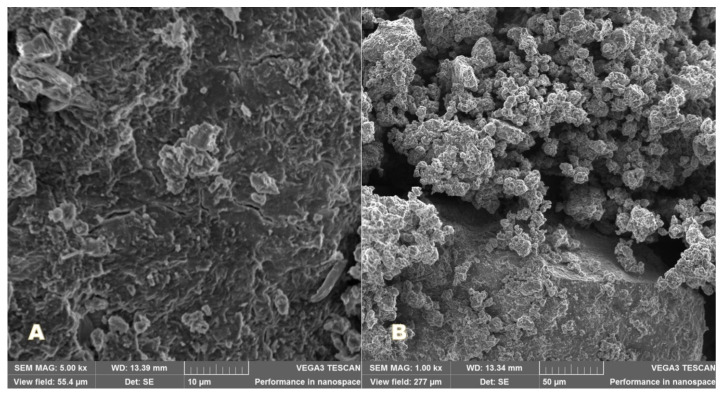
SEM image of PU particles in PU_NP_control sample: (**A**) 5000×; (**B**) 1000×.

The morphological aspect of PU_NP_acycl sample (Figure 6) and PU_NP_control sample (Figure 7) was investigated at two different magnifications: 5000× (A) and 1000× (B). The presence of more compact particles was noticed in the PU_NP_acycl sample.

### 3.8. FT-IR Spectroscopy

**Figure 8:**
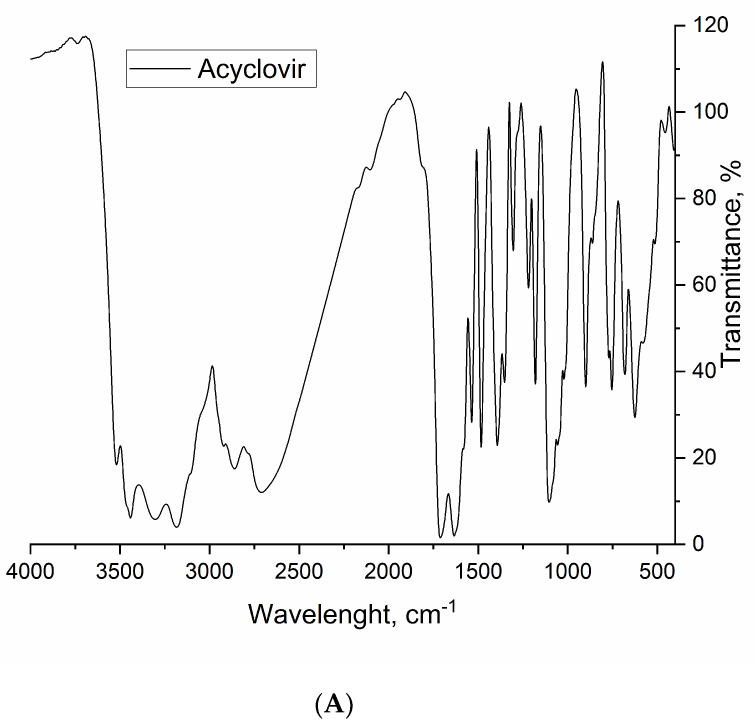

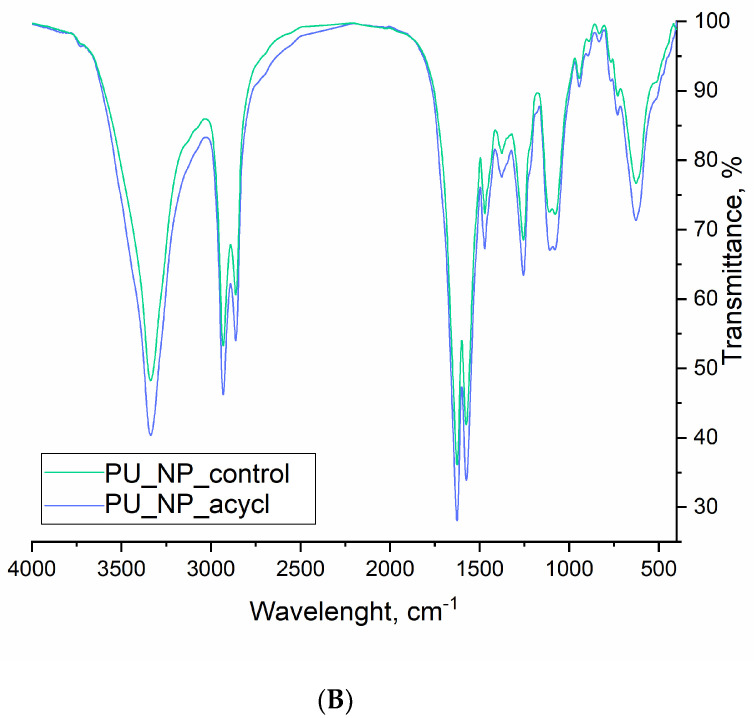
(**A**). FT-IR spectra of acyclovir. (**B**). FT-IR spectra of PU_NP_control and PU_NP_acycl samples.

The FT-IR spectra of pure acyclovir as well as the overlapped spectra of PU_NP_control and PU_NP_acycl samples are presented in Figure 8A,B, respectively.

### 3.9. SANS Analysis

**Figure 9:**
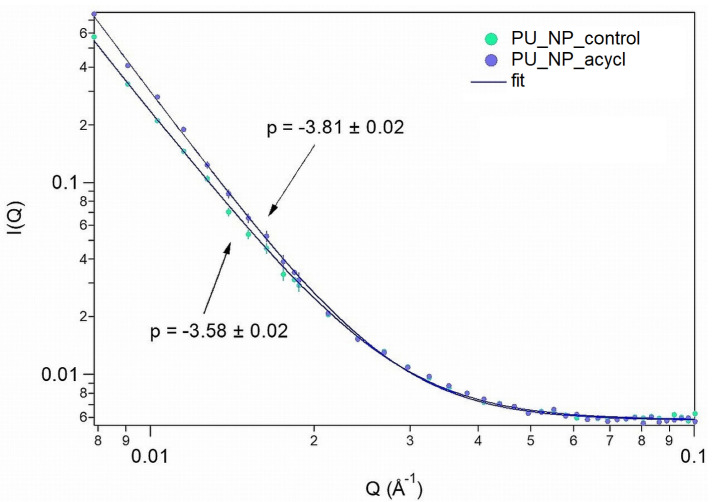
SANS curves of PU_NP_control and PU_NP_acycl samples (the continuous line represents the fitted model).

The obtained SANS curves were fitted with Equation (5) with *p* exponent values for PU_NP_control and PU_NP_acycl samples 3.81 and 3.58, respectively (Figure 9).

In the available Q (scattering vector) range no pore-size could be obtained, thus allowing the conclusion that the pores/particle sizes were larger than ~70 nm.

### 3.10. Skin Irritation Evaluations

**Figure 10:**
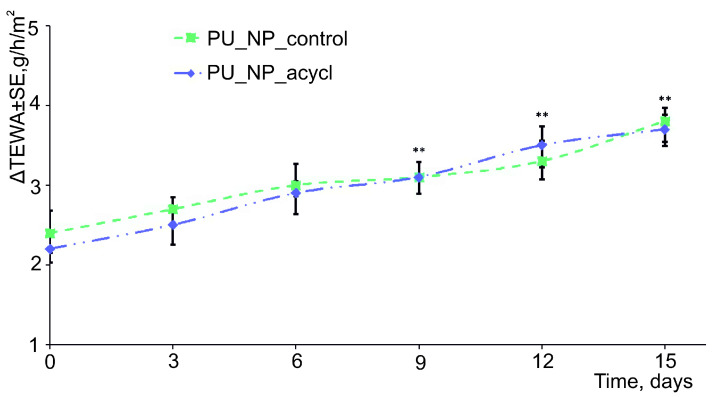
Evolution of TEWA values during the experiment.

**Figure 11:**
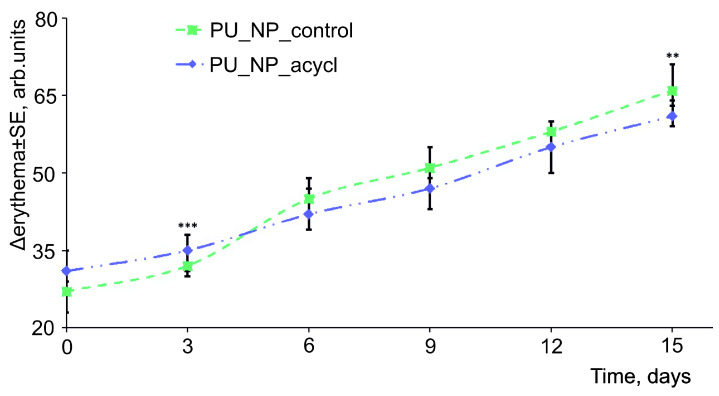
Evolution of erythema during the experiment.

Figure 10 and Figure 11 display the evolution of TEWA and erythema levels, respectively, as main indicators of the skin irritation induced by tested samples. Minor increases (between 2–4 g/h/m^2^ for TEWA and between 25–70 arb. units for erythema) were observed for the two tested samples.

## 4. Discussion

The design of new drug delivery systems based on advances in nanosciences as well as their involvement in modern medicine provide new tools able to enhance the therapeutic efficacy of numerous classic drugs. The main objectives generally pursued by the use of drug carriers consist in the protection of the loaded active agents against degradation and the reduction of systemic or local drug-induced toxicity [28]. The main applications of PU, still in continuous development, are as thermal insulations, different automotive components, elastic mattresses, adhesives, etc. Their suitability as biomedical materials started to be investigated a few decades ago by Boretos and Pierce [29], who reported their vascular acceptability in experimental heart-assist pump chambers and continued with the design and production of various cardiovascular, obstetrics and gynecology devices, reconstructive surgery materials, and more [30].

Rangasamy et al. [31] have reported the synthesis of a niosome-based carrier able to encapsulate between 45% and 84% acyclovir depending on the preparation method and the cholesterol: Span^®^ 80 ratio. Higher loading percentages were obtained when the process was conducted by hand shaking with a 1:3 cholesterol: Span^®^ 80 ratio. The 77.9% encapsulation percentage we achieved is comparable to the one reported by TLC Lima et al. [32] for hydrophobic, biocompatible and biodegradable chloroquine diphosphate-loaded nanoparticles designed as antiherpetic agents and produced by the emulsification-solvent evaporation method (64% loading efficacy).

The drug release profile in SBF medium exhibits a slight increase in the acyclovir concentration with an average change around 16% in the first week and approx. 12% in the second week of carrier nanoparticles’ degradation. This late release from the PU particles is considered appropriate for a liver-metabolized compound (plasma half-life approx. 3 h) which is also excreted in urine as unchanged drug (60–90%) [33]. The drug release from PU particles can be easily adjusted by using different polyether/polyester ratios during their synthesis which will result in final products with different degradation speeds.

A problematic issue in terms of acyclovir’s therapeutic effectiveness is its oral absorption; the process is pH-dependent and takes place predominantly in the upper gastrointestinal tract according to a previous in vivo investigation [34]. Therefore, the newly prepared PU nanoparticles loaded with acyclovir may represent a useful approach to achieve the sustained release of the drug at its absorption site and, subsequently, to prolong its activity with minimum of side effects.

The PU nanoformulations were designed to incorporate acyclovir in order to improve its dissolution profile and, subsequently, its bioavailability. Indeed, the in vitro dissolution studies revealed a dramatic solubility increase in simulated intestinal medium; in gastric medium, the solubility of the drug remains virtually unchanged. Nevertheless, a significant change in shape occurs in gastric environment indicating the sustained release of the drug. Previous studies revealed that polymer nanoparticles are able to optimize drug bioavailability in cases where it is prejudiced by the drug’s poor solubility or permeability [35]. Given its high solubility in acid medium and its pKa values (2.27 and 9.25) [36], acyclovir should prolong its residence at gastric level and be subjected to a sustained release in order to allow the overall sustained drug oral absorption and, subsequently, to improve its systemic bioavailability. The acyclovir-loaded PU particles were able to provide the drug’s sustained release over 24 h in both tested media. However, only the simulated gastric medium revealed a dissolution profile that fits the literature parameters which define this type of delivery systems: 10–30% cumulated drug release in the first 2 h, 50% drug release in 8 h, and 80% drug release over 24 h [37]. The cumulated drug release in the simulated intestinal medium was lower than the above-mentioned parameters, presumably due to the pH-dependent acyclovir solubility. Natural polysaccharides were used by Soni et al. in order to induce the sustained release of acyclovir; the authors reported a significant amount of 85–97% drug release over 720 min [38]. A gastroretentive system for acyclovir was designed by Shin et al. in 2019 who revealed a sustained release of the drug and a prolonged absorption as a result of a 12 h gastric residence of the delivery system [39]. Our results are also consistent with those reported by Stulzer et al. who prepared polymer microparticles loaded with acyclovir which provided the sustained release of the drug in both intestinal and gastric media, with higher dissolution rates in the acidic environment [40]. Therefore, we may state that the in vitro dissolution results confirm some previously reported data; also, the acyclovir-loaded PU nanoparticles are able to achieve the sustained drug release following oral administration thus qualifying as potential future therapeutic options. The significant difference between the cumulative drug release percent obtained in the intestine and gastric-like medium compared with SBF is important to appreciate the enhanced oral absorption and oral bioavailability.

In vitro models based on artificial membranes, typically used in penetration experiments, are designed to evaluate the capacity to transfer active agents through skin layers, gastrointestinal tract mucosa and cell membranes [41]. This assay has revealed that half of particles penetrated the membrane in the first ten hours, and these are similar results compared to other nanoparticles tested as drug delivery systems on PVDF membranes [42].

The thermal stability of PU was previously described by C Bolcu and F Borcan [15] who reported that PU materials can be heated up to 300 °C without the degradation of the macromolecular chains. No significant peaks can be noticed in the thermograms of PU_NP_control and PU_NP_acycl samples both containing PU nanoparticles (Figure 5); the absence of the drug’s characteristic peak around 255–260 °C on the PU_NP_acycl sample curve indicates the complete encapsulation of acyclovir inside the PU carrier nanoparticles. The glass transition of PU materials cannot be seen on the thermograms due to the fact that, according to P. Somdee et al., the process occurs between −57 °C and −52 °C [43].

The polydispersity index (PDI) is a parameter which describes sample heterogeneity and may vary between 0.0 (perfectly homogeneous samples) and 1.0 (highly heterogeneous samples). Our newly synthesized PU_NP_acycl nanoparticles belong to a single population with enhanced homogeneity (around 78 nm) while the PU_NP_control sample displays two particles’ size populations: a majority nanoparticle population with an average diameter of 91 nm and a smaller population with an average size of 214 nm. Several advantages of drug delivery systems based on single-populational particles, as our PU_NP_acycl sample, are presented in literature [44,45]: increased particles stability, encapsulation efficiency, drug release profile, and bio-distribution.

The medium stability of these samples against the nanoparticle tendency to form clusters was confirmed by the Zeta potential values as well as the SEM images. The most unstable particles show Zeta potential values of around 0 mV while Zeta potential values outside the −30 mV to +30 mV range are specific to highly stable systems [46]. The PU nanoparticles synthesized in the current work show Zeta potential values ranging between +26.9 and +29.1 mV thus showing medium to high stability; these results are highly related to PDI values that were recorded for these particles.

Table 2 comparatively describes the most relevant peaks obtained by FT-IR spectroscopy on pure acyclovir, PU_NP_control and PU_NP_acycl samples, respectively. One can notice numerous differences between the pure acyclovir spectrum and the spectra of PU_NP_control and PU_NP_acycl samples, respectively, which can be attributed to the shielding of the pure drug due to its entrapment inside the PU nanoparticles; additionally, the PU_NP_control and PU_NP_acycl samples display a high degree of similarity which indicates that acyclovir is not chemically bound to the walls of the PU nanoparticles. Similar behaviors have been presented in other studies containing FT-IR spectroscopy on a PU carrier [19,47].

The analysis of the molecular architecture as well as the morphology, size and composition of potential aggregates can be conducted directly by SANS studies which provide a high contrast due to the use of protiated organic excipients [48]. The PU_NP_control and PU_NP_acycl samples were compared in terms of nanostructure organization by using the SANS method.

The obtained *p* exponent in the large Q-range characterizes the interfaces present in the sample, namely the pore-particle surface. The *p* exponent values for porous structures such as ionogels or aerogels [49] usually vary between 2 and 4, 4 being specific to smooth interfaces, while values between 3 and 4 correspond to fractal-like, rough surfaces. Values between 2 and 3 characterize mass fractal systems. The higher fractal exponent for the PU_NP_acycl sample indicates a smoother pore/particle surface, evidenced as well by SEM measurements at micrometric level. The addition of acyclovir was able to slightly restructure the PU particles so that the more compact, nanometric particles formed a loose, porous structure, similar to the one of pure PU (Figure 6 and Figure 7). The drug delivery systems based on porous particles have the following advantages [50]: stable uniform porous structure, high surface area, tunable pore size and well-defined surface properties.

Tests of the newly synthesized chemicals on animal or human skin are often used due to their high sensitivity for detecting potential skin toxicity. Nowadays, reliable and reproducible non-invasive techniques are used to analyze the most important skin parameters (melanin, erythema, transepidermal water loss (TEWA), hydration, pH, sebum, etc.) in order to conduct various dermatological and cosmetic assessments.

O Kose et al. [51] have reported that modifications of the skin parameters (in particular the level of TEWA and erythema) after the application of an irritative agent are fast and directly proportional with the irritation potential of the respective chemical agent. In the current 15-days experiment, the evolution of TEWA was characterized by an increase of 0.7 g/h/m^2^ in the first week. A similar value was determined in the second week of the experiment for both samples. Usually, an increase of the TEWA values is associated with a decrease of the skin barrier function. However, very low values of TEWA variations were recorded thus indicating the tested samples as reliable chemical agents [52]. The assessment of erythema values showed a daily increase of about two units in the first week and approx. three units/day during the second week of the experiment for both samples. A definite irritative process was already evaluated and established as reference in one previous study conducted in our Phyto-science Research Center. Briefly, the repeated application of pure chili pepper extract on human skin has increased the erythema level by approximately 100 units over two weeks [14]. Therefore, we may conclude that the erythema values recorded for the PU tested samples indicate their low irritative potential therefore their suitability for topical application. PU materials are described as safe, biocompatible, and non-irritant in literature [53]. On the other hand, D. Ciorba et al. [54] has reported important TEWA modifications (around 30 g/h/m^2^) in a study based on a test with 100 μg/L chloroform solutions and the amplitude of these changes can be considered as an irritation reference.

## 5. Conclusions

Polyurethane nanoparticles have been synthesized by using a polyaddition process between a mixture of two aliphatic isocyanates and a mixture of an aliphatic diol and ether polyol as main raw materials. The samples of acyclovir-loaded and unloaded PU nanoparticles were investigated in terms of encapsulation efficacy, drug release profile, FT-IR spectra, SEM, SANS, thermal analysis, and in vivo skin irritation. The results emphasized the suitability of these particles for the transmembrane transfer of the antiherpetic agent.

To the best of our knowledge, there are currently only a few studies focused on the potential use of polyurethane nano- and micro-particles as drug delivery systems. In addition to the novelty of this investigation, the inexpensive raw materials, the biocompatibility of the final products, the easy pathway to synthesize particles with various sizes depending on the chain extender amount, as well as the facile modulation of the drug release profile through the variation of the ester/ether polyol ratio are advantages of huge significance for the use of polyurethanes as drug delivery systems. Therefore, the experimental results reported in the current paper recommend further investigations on PU nano- and microparticles as well as their loading with active drugs. Future studies could lead to the design of PU drug delivery systems sufficiently effective in vitro and in vivo to become the subject of clinical trials.

## Figures and Tables

**Table 1:** Zetasizer characterization of synthesized samples.

Sample	Size, nm (Signal Intensity)	PDI ^1^	Zeta Potential, mV
PU_NP_acycl	78 ± 4 (100%)	0.2	+29.1 ± 3.4
PU_NP_control	91 ± 9 (82%) 214 ± 11 (18%)	0.5	+26.9 ± 2.1

^1^ polydispersity index.

**Table 2:** Interpretation of FT-IR spectra.

Wavenumber, cm^−1^	Functional Group	Presence in Sample	
		Acyclovir	PU_NP_acycl/PU_NP_control
3520–3440, b	Dimeric O-H stretch^32^	yes	no
3340–3335, s	N-H stretch^10,32^	no	yes
3300–3180, b	O-H stretch^32^	yes	no
2930–2660, s	Methyl C-H asym/sym stretch^32^	no	yes
2860–2710, b	Methoxy C-H stretch^32^	yes	no
1710–1630, b	Ring C=O stretch^10^	yes	no
1625–1575, s	Urethane N-H deformation^10^	no	yes
1535–1470, s	Methylene C-H bend^32^ N-H deformation^10^	yes	yes
1390–1350, b	Aromatic C-N stretch^32^	yes	no
1255 + 940–730 w, s	C-O- stretch^32^	no	yes
1220–1180, s	Tertiary amine C-N stretch^32^	yes	no
1110–1075, b	Alkyl ether C-O-C stretch^32^	yes	yes
770–625, s	Skeletal C-C vibrations^32^	yes	yes

Abbreviations: b, broad; s, sharp; w, weak.

## Data Availability

Data sharing not applicable.
